# Intraoperative gaze guidance with mixed reality

**DOI:** 10.1049/htl2.12061

**Published:** 2023-12-13

**Authors:** Ayberk Acar, Jumanh Atoum, Amy Reed, Yizhou Li, Nicholas Kavoussi, Jie Ying Wu

**Affiliations:** ^1^ Department of Computer Science Vanderbilt University Nashville Tennessee USA; ^2^ Department of Urology Vanderbilt University Medical Center Nashville Tennessee USA; ^3^ Department of Electrical Computer and Systems Engineering Case Western Reserve University Cleveland Ohio USA; ^4^ Present address: Department of Computer Science Vanderbilt University Nashville Tennessee USA

**Keywords:** augmented reality, biomedical communication, gaze tracking

## Abstract

Efficient communication and collaboration are essential in the operating room for successful and safe surgery. While many technologies are improving various aspects of surgery, communication between attending surgeons, residents, and surgical teams is still limited to verbal interactions that are prone to misunderstandings. Novel modes of communication can increase speed and accuracy, and transform operating rooms. A mixed reality (MR) based gaze sharing application on Microsoft HoloLens 2 headset that can help expert surgeons indicate specific regions, communicate with decreased verbal effort, and guide novices throughout an operation is presented. The utility of the application is tested with a user study of endoscopic kidney stone localization completed by urology experts and novice surgeons. Improvement is observed in the NASA task load index surveys (up to 25.23%), in the success rate of the task (6.98% increase in localized stone percentage), and in gaze analyses (up to 31.99%). The proposed application shows promise in both operating room applications and surgical training tasks.

## Graphical abstract

Efficient communication and collaboration are essential in the operating room for successful and safe surgery. In this work, to improve communication and collaboration, mixed‐reality headsets is used to share gaze markers between expert and novice surgeons for intraoperative guidance. In the experimental setup, improvements are observed in task success, NASA TLX metrics, and gaze analyses.

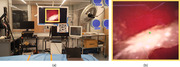

## 1 INTRODUCTION

Augmented reality (AR), virtual reality (VR), and mixed reality (MR) have the potential to transform the workflow in many fields, including the operating room (OR) experience [1, 2]. These advances can help surgeons collaborate on different tasks remotely, decrease their mental and physical loads, support surgical training and skill assessment, and facilitate communication in the operating room. While VR technologies offer an immersive experience, their use in the OR is limited due to the risk of malfunction and the lack of a see‐through display. On the other hand, optical see‐through MR and AR technologies with built‐in fail‐safe design can be adapted into the flow of surgery, and surgeons can use them with ease with minimum risk to improve their normal interaction. In the case of failure, surgeons can continue the operation without an interruption to their direct vision of reality [3].

Endoscopic surgeries are potential use cases for AR and MR technologies. Navigating the 3D inner structure is done in such surgeries using an endoscope and 2D surgical monitors. Endoscopic navigation creates a challenge by complicating the surgical instructions for surgeons, in both training and intraoperative processes guided by experts. Navigation, indication of areas of interest, and guiding novice surgeons become harder with surgical monitors compared to direct visual contact [4]. In the commonly seen case of kidney stone surgeries, these limitations can increase the risk of missing stones, leading to subsequent stone events and re‐operation. According to Scales Jr et al., approximately 1 in 11 people in the USA suffer from kidney stones [5] and around 23% of the patients that undergo operation require another intervention in less than 20 months period [6], indicating missed stones during the first operation.

In this study, we propose an MR application for expert surgeons to share their gaze with novices to improve their communication, using Microsoft HoloLens 2 headsets (Microsoft Corporation, Redmond, WA). This application allows them to guide and interact with decreased effort and higher accuracy, compared to standard verbal communication. The application can record and share the gaze to assist during the operation and surgical training. The recordings can be used in post‐operation skill assessment as well. We evaluate the efficacy and efficiency of our proposed method with a user study of ureteroscopy on kidney phantoms conducted with urology expert and novice surgeons at Vanderbilt University Medical Center.

## 2 RELATED WORK

### 2.1 Extended reality (XR) in the operating room

In the medical field and in operating rooms, AR and MR equipment are used for preoperative planning, execution, and postoperative skill assessment in different areas such as paediatric surgery [7], orthopaedic surgery [8], tumour surgery [9], and neurosurgery [10]. Sanchez‐Margallo et al. created an application that uses MR for training and surgical planning [11]. In a narrative literature review, Vervoorn et al. collected and analyzed 57 papers about MR in the operating room and concluded that the overall experience is positive despite the limitations of head‐mounted displays [12]. In a feasibility study with 25 orthopaedics surgeons, Dennler et al. found that on a 100‐point scale, surgeons rated overall satisfaction with AR in the OR as 78 ± 15 and demand for future access as 75 ± 22 points [13].

While these studies indicate the demand for XR and specifically MR technologies, they mostly focus on hologram manipulation, planning, and remote connections. In a study by Garosi et al., 63% of the surgeries had at least one concerning communication pattern, and 32.69% of the communication patterns in the OR were possibly associated with poor performance [14]. Current modes of interaction are open to improvement and there is an unmet need in using XR to facilitate communication in the OR.

### 2.2 Gaze applications in surgery

Eye tracking and gaze analysis in the operating room are used for environment interaction, device controls, and skill assessment and classification. Ezzat et al. evaluated “an eye‐tracking based robotic scrub nurse” and concluded its performance is acceptable to test users [15]. Kogkas et al. used wearable eye‐tracking technologies and advanced computer vision methods in their study to allow perceptually enabled interactions in the OR [16]. To demonstrate eye‐tracking's possible functionalities, they projected surgeon fixation points to the environment using a laser controlled by a robotic arm. While this study explored the possibilities of a framework that collects and uses information from different media, the use of MR headsets has the potential to further simplify such tasks. Yu et al. uses gaze and viewing patterns to complete a task analysis in microsurgery [17].

The use of gaze metrics to evaluate the skills of the surgeons and classify surgeons as experts or novices are also popular study areas in eye‐tracking‐based applications [18, 19, 20, 21, 22]. Our previous work showed that gaze metrics such as fixations and area of interest are significant metrics of expertise in kidney stone identification task [21]. In addition to these studies proving the significant distinction between the gaze pattern of expert and novice surgeons, Vine et al. showed that employment of expert gaze strategies by novices can improve the performance and learning experience [23]. Liu et al. indicated that with behavioural training, trainees reach equivalent performance to the expert surgeons but their gaze patterns are still not as efficient as them, and suggested involving eye tracking technology in training programs [24]. In an anonymous survey conducted by Marín‐Conesa et al., 83% of participants consisting of medical students, practicing doctors, medical teachers, and medicine residents agreed with the usefulness of eye‐tracking glasses in the OR for communication and teaching purposes [25]. Overall, related studies advocate for the employment of eye tracking in surgical training and implementation flows.

### 2.3 Communication and collaboration

Studies related to gaze‐based communication mainly focus on remote collaboration in different domains. Brennan et al. evaluated the efficiency of gaze sharing on a collaborative search task using two monitors and revealed that compared to solitary search, gaze‐shared search was twice as fast and efficient [26]. Similarly, Lee et al. observed a significant improvement in collaboration during a video conference with a gaze‐sharing system [27]. Bai et al. and Kim et al. created systems for remote collaboration that employs hand and eye movements, and showed that gaze markers improve communication compared to verbal interaction; however, they both stated that users prefer the methods that combine gaze and hand movements [28, 29]. Jing et al. incorporated gaze behaviours into the remote interaction in addition to the normal gaze‐sharing process [30]. In another study, Jing et al. compared different types of gaze visualizations and the effect of gaze behaviours in a co‐located environment with AR head‐mounted displays and showed that gaze markers are helpful indicators for intentions and joint attention [31]. Erickson et al. analyzed the effectiveness of gaze rays on target identification with a simulated gaze and examined the error types [32].

The number of studies on local gaze‐based collaboration using holograms is limited. To the extent of our knowledge, this is the first study to implement and test gaze sharing in a medical domain and in a mock operating room setting.

## 3 METHODS

We use HoloLens 2 eye tracking and multi‐user collaboration capabilities to share the gaze in a unidirectional way, from experts to novices. We build a Unity (2021.3.4.f1) (Unity Technologies, San Francisco, CA) application using Mixed Reality Toolkit 2 (MRTK2). We follow a similar approach to official Microsoft tutorials^1^ for multiple user applications where a shared playground is created and existing user positions are indicated with spheres; however, we replace user head positions with gaze markers in the sharing process. The application detects and tracks a user's eye gaze, creates a marker for the point of interest, and shares this marker with the other user. The other user can see and track the gaze marker and understand mentioned objects or areas using this assistance.

Before starting the application, the HoloLens 2 headset requires an eye calibration process for first‐time users. After the user puts on the headset, a prompt to start the calibration appears and the calibration application starts. The user needs to follow holograms with their eyes only, which makes the process suitable for any environment. Calibration information is stored for each user and users do not need to calibrate each time they use the device. After the calibration, users can start our application from the virtual menu.

**FIGURE 1:**
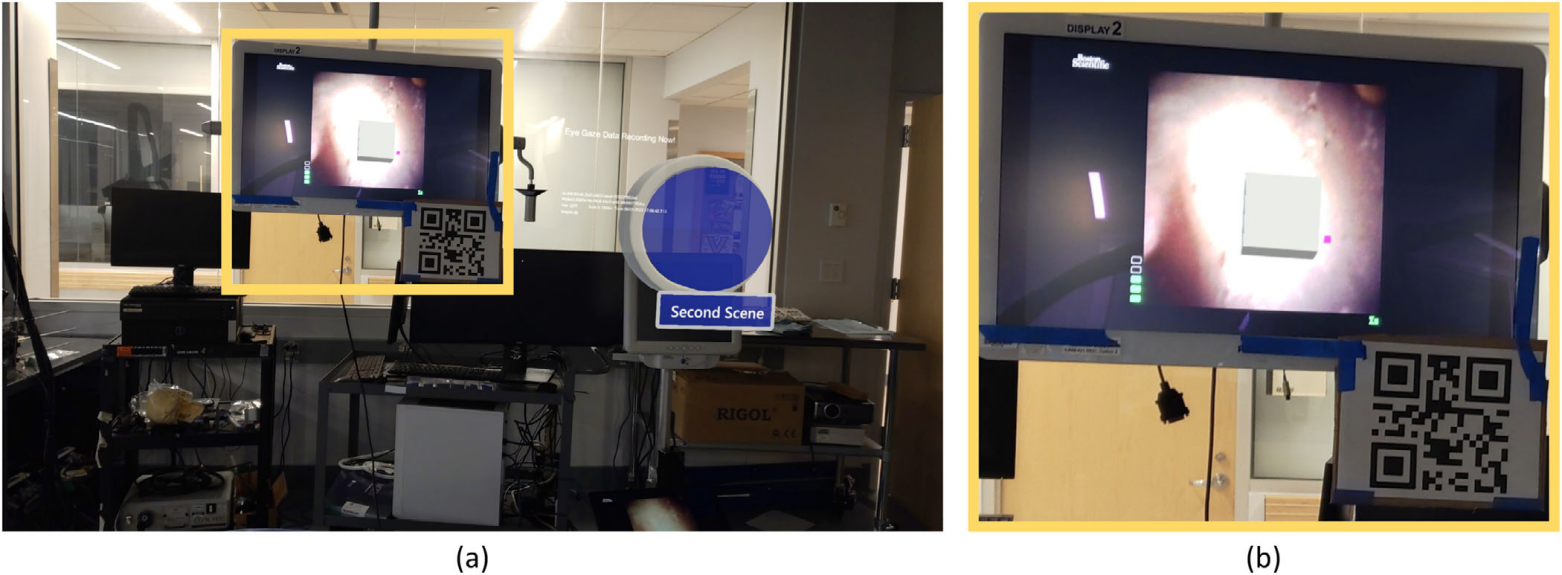
(a) The first scene of the application where QR code reading and anchor placement happens. Users can proceed to the second scene by pressing the virtual button. (b) Close‐up of the anchor position represented by the white cube. The zoomed area is indicated in the yellow square in (a).

Our application consists of two scenes. In the first scene, we create a shared virtual plane that overlays the surgical monitor and place it based on a QR code located in the environment. After the detection of the QR code and placement of the virtual plane, an anchor object is placed in the center of the mentioned plane (Figure 1). This anchor object acts as a shared center and coordinate system between two user systems.

**FIGURE 2:**
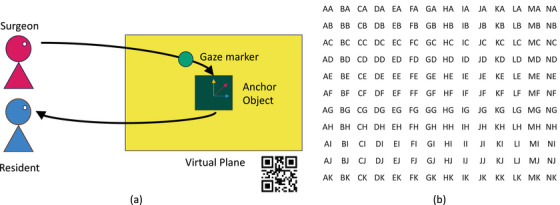
Application schematic and grid sheet. (a) Workflow of the application. Gaze marker coordinates are transformed from the user system to the shared system, and back to the other user. (b) Grid sheet of 2 cm spaced elements for gaze sharing accuracy test. Accuracy is evaluated by a verbal cross‐check by the expert surgeon fixating on a grid element and the resident surgeon reading the chosen element.

The application transfers the coordinates of an object that a user creates first into this common system, then back to the coordinate plane of the other user (Figure 2(a)). It stores and recalls anchor objects from the azure spatial anchors system. While this system supports the creation and sharing of anchors without a QR code, we observed higher accuracy for sensitive tasks like surgical operations with the assistance of QR codes. Following the anchor placement, the user can press the button for the second scene which stops the QR code detection to decrease hardware load and starts the gaze sharing process.

**FIGURE 3:**
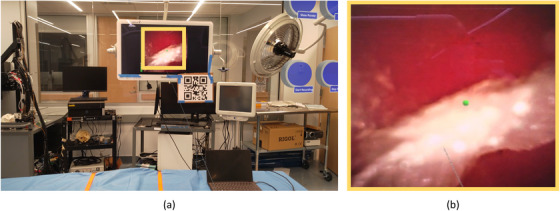
(a) The second scene of the application where the gaze of the surgeon is represented by a green marker. Buttons in this scene can be used to show/hide the gaze marker and start/stop the gaze recordings. (b) Close‐up of the marker position and the area indicated by the yellow square in (a).

In the second scene of the application (Figure 3), we use photon unity network (PUN) to share transformed gaze marker holograms. PUN sharing allows real‐time application with insignificant delays. After moving on to the second scene, we test the accuracy and measure any offset due to the limitations of the system by asking the users to do a cross‐check on grid elements (Figure 2(b)). We either show the grid sheets on the surgical monitor or print and place them in the environment, similar to the QR code. Once we verify the accuracy of the gaze sharing, the user can proceed with setting up the level of guidance for the task. The application allows users to turn on and off gaze markers and gaze recording with voice commands and buttons. Even though this application supports bidirectional sharing as well, based on the feedback from the expert and novice surgeons, we limited its capabilities to unidirectional from the expert surgeon to the novice in order to avoid any confusion.

For the gaze analysis, we use the metrics with the most significant statistical results in our previous work [21], namely total distance travelled by eye gaze, fixation time, and gaze area in the area of interest (AOI) [21]. The total distance travelled by eye gaze measures all movement on the virtual plane. We use the same definition of fixation, which is limited eye movement for at least 300 ms in a circle with 1.5 cm diameter, and overall fixation time is the sum of time spent in each fixation period. Finally, we set the AOI to be the part of the surgical monitor displaying the endoscopic video stream, with a size of 35.5 cm × 35.5 cm.

## 4 EXPERIMENTAL SETUP

**FIGURE 4:**
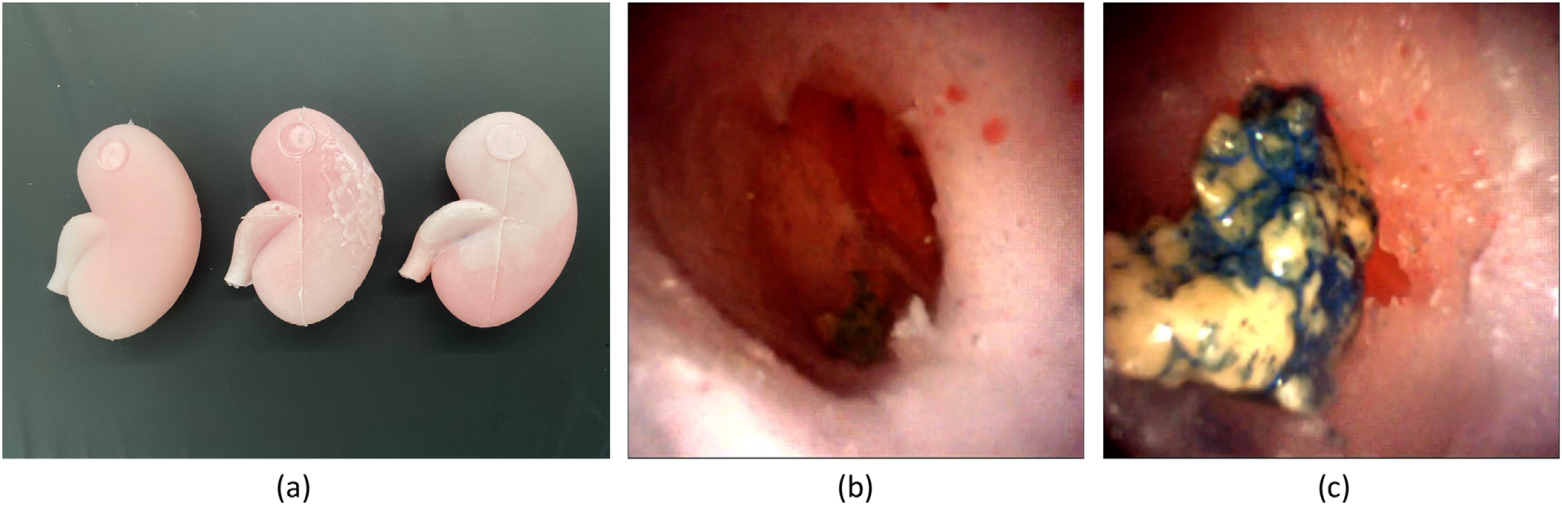
(a) Outer structure of kidney phantoms.(b) The inner structure of phantoms from endoscope camera. (c) Example of detected stone in ureteroscopy process.

**FIGURE 5:**
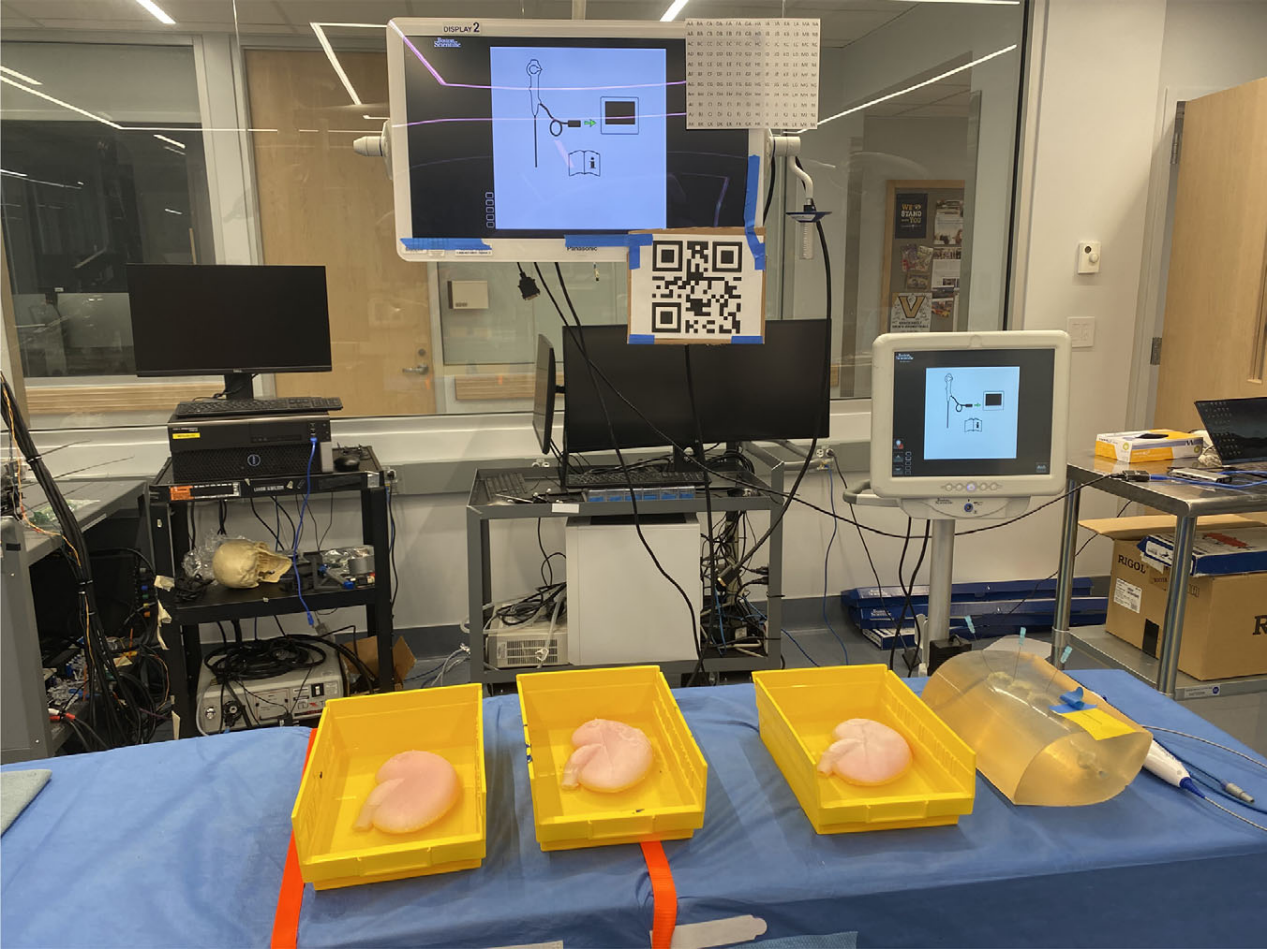
Experimental setup with QR code and accuracy measurement grid sheet placed on the surgical monitor, three kidney phantoms used in the experiment submerged in water, and a warm‐up phantom.

To evaluate our proposed application, we conducted a user study with 10 urology residents and fellows who perform fewer than 100 ureteroscopies a year, and 2 expert surgeons (both fellow‐trained in kidney stone disease and performing more than 100 ureteroscopies a year). For this study, we prepared three kidney phantoms by casting silicon using 3D‐printed kidney collecting systems from CT scans, and an outer mould. Similar to the implementation of Adams et al. [33], we placed the collecting system inside the outer mould, filled the mould, then melted the inner structure to obtain a hollow, kidney‐like structure. Expert surgeons validated the complexity and exploration difficulty of the renal collecting systems in each phantom as similar. We also prepared kidney stone models from Begostone [34] and dyed them in different colours to keep track of detected stones during ureteroscopy (Figure 4). Stones were placed into the different parts of the phantoms by the expert surgeons using a basket. The experimental setup with all phantoms and OR environment is shown in Figure 5.

In each trial, similar to the intra‐operative setting ureteroscopy, residents aim to explore the kidney phantom submerged in water using LithoVue ureteroscopes (Boston Scientific, Marlborough, MA) and report the number of stones they found. Novices perform ureteroscopy three times, each with one of the following involvements of the expert surgeon: no assistance (NA), verbal assistance (VA), or gaze assistance (GA). On NA tasks, residents complete the task without guidance while the expert surgeon watches. On VA tasks, expert surgeons guide residents as they do in current practice in operating rooms, by verbal interaction and gestures. On GA tasks, our application is used alongside verbal assistance. Before the start of any task, users are able to warm up using a different phantom that is not used for the rest of the study. For each user, the order of tasks, phantom numbers, and guiding surgeon are determined randomly to avoid any bias (Table 1).

**TABLE 1:** Number of users for each possible test scenario.

		1st task	2nd task	3rd task
	Phantom 1	2	2	0
No assistance	Phantom 2	1	0	1
	Phantom 3	0	2	2
	Phantom 1	1	1	1
Verbal assistance	Phantom 2	1	1	1
	Phantom 3	2	1	1
	Phantom 1	1	1	1
Gaze assistance	Phantom 2	2	1	1
	Phantom 3	0	1	2

The novice surgeons can stop the operation when they are satisfied with their progress and think they are done. Expert and novice surgeons wear HoloLens 2 headsets through each task, to record gazes for post‐analysis and use gaze‐sharing assistance when needed. After the completion of each task, residents fill out a NASA task load index (NASA‐TLX) form [35]. The form contains six categories: mental demand, physical demand, temporal demand, performance, effort, and frustration; rated between 1 (low) and 20 (high). We evaluate and compare task completion time, the number of stones found, survey responses, and gaze skill assessment metrics for each user and task.

## 5 RESULTS AND DISCUSSION

### 5.1 Performance

Table 2 shows the mean user performance and NASA‐TLX test scores for all three task groups: NA, VA, and GA. GA has the highest stone identification percentage with 92%, followed by verbal assistance (86%) and no assistance (80%). These values indicate a significant improvement over the standard practice of verbal interaction. Our method shows the potential to increase the operation success rates and decrease missed kidney stones which result in re‐operations.

**TABLE 2:** Mean user performance and NASA‐TLX survey results. Standard deviations are given in parentheses. For NASA‐TLX metrics: 1‐very low 20‐very high (except performance, 1‐perfect 20‐failure). Bold values show the best performance in the category. Note that one user did not fill out the survey after the gaze assistance task.

	No assistance	Verbal assistance	Gaze assistance
Percentage of stones	80 (18.9)%	86 (21.2)%	**92 (10.3)%**
Completion time (s)	**225.5 (130.3)**	228.6 (148.3)	238.2 (136.6)
Mental demand	12.4 (3.4)	10.7 (3.8)	**8.0 (4.1)**
Physical demand	9 (3.6)	8.4 (3.4)	**7.7 (3.1)**
Temporal demand	8.3 (4.5)	6.8 (3.5)	**6 (3.2)**
Performance	10.6 (4.0)	9.7 (4.5)	**9.0 (4.2)**
Effort	13.0 (1.9)	12.2 (1.3)	**11.9 (3.2)**
Frustration	10.4 (4.5)	8.1 (3.4)	**7.7 (4.7)**

No assistance tasks have the shortest task completion time, followed by VA and GA, which can be associated with unfamiliarity with the system. Novice users have experience ranging from year‐one residents to year‐one fellows. We observe that with GA, less experienced novices have decreased completion time compared to NA or VA. However, novices with relatively more experience have longer task durations, affecting the average. Even though we do not see any improvement in the completion time, the overall stone identification percentage increases in this user group as well. Based on this observation, introducing the application in an early stage of surgeon training may be more beneficial for learning the correct techniques. Overall, novices with different experience levels showed improvement in task success rate and had a better experience based on other metrics.

### 5.2 Usability

With our proposed method, we observe an improvement over VA in every category of the NASA‐TLX survey (Table 2). The highest improvement of GA over VA is in mental demand with a 25.23% decrease. Temporal demand, where the users are asked about their discomfort related to the pace of the task, follows it with an improvement rate of 11.76%. Physical demand is decreased by 8.33% and performance metric is improved by 7.22%. The rest of the improvements are listed as frustration (4.94%) and effort (2.46%). Relatively smaller improvement percentages for those two categories may be due to the users' unfamiliarity with the technology.

Users commented that the gaze marker was very helpful in giving directions and reduced the necessity of verbal commands. One user mentioned that gaze assistance was especially helpful with small movements and another one claimed that they can better focus on their hand technique and wrist movements rather than the commands. We did not get any negative comments about our application from the users.

Overall, we did not find any significant negative effects of our application on the surgical procedure; on the contrary, it improves the surgeons' experiences in categories that are widely accepted as standard.

### 5.3 Eye gaze metrics

We measure three gaze metrics of expertise level, as defined in the methods section: 1) the total distance travelled by eye gaze, 2) total fixation time, and 3) the gaze area in AOI. We expect expert surgeons to have a more condensed gaze area in AOI, with less distance travelled and higher fixation duration.

Table 3 shows the results of gaze analysis. Note that the gaze analysis is done with five novice surgeons and the accompanying/guiding expert surgeons for each of them. We discarded the data from users that did not start the recording for a task, and the ones with unstable tracking for analysis due to problems such as glasses glare or false calibration. All users included in the table have complete data for each task.

**TABLE 3:** The average gaze measurement metrics for both expert and novice surgeons under three guidance systems. Standard deviations are given in parentheses.

Task	Participant	Total distance (cm) ↓	Fixation time (s) ↑	Area in AOI (cm 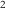 ) ↓
No assistance		3315.92 (1618.20)	146.45 (17.96)	1352.13 (557.95)
Verbal assistance	Novice	3304.14 (1086.84)	141.77 (31.17)	1214.27 (553.47)
Gaze assistance		**3117.73 (1162.36)**	**187.12 (44.18)**	**1091.33 (505.34)**
No assistance		3035.47 (1168.54)	162.89 (17.13)	1151.39 (477.95)
Verbal assistance	Expert	2761.98 (373.67)	151.30 (32.20)	966.39 (588.10)
Gaze assistance		**1953.62 (319.70)**	**202.53 (38.18)**	**928.262 (605.49)**

According to the results, compared to VA standard procedure, novices have 31.99% longer fixation duration in GA tasks, indicating a higher level of expertise. With GA, their gaze is more condensed in AOI, with a decrease of 10.12% from the area in the VA tasks and they have 5.64% shorter total gaze traveling distances over VA. With our application, we observe an increase in performance in all statistically significant metrics. These improvements display a more efficient gaze performance over other guidance methods.

### 5.4 Future work and limitations

In our future work, we plan to test the gaze‐sharing capabilities of the application in the whole OR and not only on the surgical monitor. We also plan to extend the communication to other members of the surgical team. In addition, we plan to incorporate hand movements and hand‐controlled markers to further improve the delivery.

One limitation we observe in our application is the limited accuracy due to factors such as HoloLens capabilities and initial position differences. These problems can be solved by additional calibration processes. In addition, the HoloLens cannot simultaneously read QR codes and share the holograms due to limited processing capabilities. Therefore, the QR code is only read in the first scene and replaced by a spatial anchor. This may result in decreased accuracy over time, depending on the anchor modules' performance to preserve the position in the real world.

In the clinical environment, surgeons' unfamiliarity with XR devices may cause decreased performance in the adaptation period. While see‐through displays allow surgeons to continue operating in case of a malfunction and do not require additional sterilization, virtual buttons can be unintuitive for inexperienced users. In future work, we plan to test our application in an actual operation and collect feedback from surgeons. For clinical use, we can extend the application to display additional information about the surgery or the condition of the patient.

Our application uses off‐the‐shelf sub‐modules, which are proven in performance but not specifically designed for medical scenarios. While the use of these sub‐modules limits the technical novelty, for the scope of this project their performances were sufficient. Future work may include improvement of the modules with better use of surgical monitors or additional tools from the OR. One improvement we are developing is a pipeline for automatic detection of the area of interest rather than manually setting for each operating room.

Finally, we recognize that the number of participants is not enough to perform a significant statistical assessment and overcome the reasons such as learning bias that may affect the results. Even though we completed our user experiments in a randomized way, better‐balanced trials can be done with more users. Users from independent institutions can have more objective comments. Our future work involves conducting a more comprehensive user study with more users. In addition, future work may focus on the effect of eye gaze‐based training over longer periods of time.

## 6 CONCLUSION

In conclusion, we propose a gaze assistance system for assisting intraoperative communication. We can use the system for improving surgical training workflows. We conducted a user study to evaluate different levels of assistance during the phantom kidney stone identification task. We show that gaze guidance improves the surgeon experience and increases the percentage of stones detected by 6.98%. This gaze guidance system can lead to improved performance by trainees, leading to fewer missed kidney stones, and better clinical care. It has the potential to transform the OR and can be extended to improve communication with the rest of the surgical team on different tasks.

## AUTHOR CONTRIBUTIONS

**Ayberk Acar**: Conceptualization; data curation; formal analysis; methodology; project administration; software; writing—original draft; writing—review and editing. **Jumanh Atoum**: Data curation; formal analysis; methodology; software; validation; writing—original draft; writing—review and editing. **Amy Reed**: Conceptualization; data curation; investigation; resources; supervision; validation. **Yizhou Li**: Resources; software. **Nicholas Kavoussi**: Conceptualization; data curation; funding acquisition; investigation; project administration; supervision; validation; writing—original draft; writing—review and editing. **Jie Ying Wu**: Conceptualization; funding acquisition; investigation; methodology; project administration; supervision; writing—original draft; writing—review and editing.

## CONFLICT OF INTEREST STATEMENT

The authors declare no conflicts of interests.

## Data Availability

The data that support the findings of this study are available on request from the corresponding author. The data are not publicly available due to privacy or ethical restrictions.

## Footnotes

1
https://learn.microsoft.com/en‐us/windows/mixed‐reality/develop/unity/tutorials/mr‐learning‐sharing‐01
